# The Impact of H_2_S on Obesity-Associated Metabolic Disturbances

**DOI:** 10.3390/antiox10050633

**Published:** 2021-04-21

**Authors:** Ferran Comas, José María Moreno-Navarrete

**Affiliations:** 1 Department of Diabetes, Endocrinology and Nutrition, Institut d’Investigació Biomèdica de Girona (IdIBGi), CIBEROBN (CB06/03/010) and Instituto de Salud Carlos III (ISCIII), 17007 Girona, Spain; fcomas@idibgi.org; 2 Department of Medical Sciences, Universitat de Girona, 17003 Girona, Spain

**Keywords:** H_2_S, obesity, adipose tissue, diabetes, insulin, NAFLD, CVD

## Abstract

Over the last several decades, hydrogen sulfide (H_2_S) has gained attention as a new signaling molecule, with extensive physiological and pathophysiological roles in human disorders affecting vascular biology, immune functions, cellular survival, metabolism, longevity, development, and stress resistance. Apart from its known functions in oxidative stress and inflammation, new evidence has emerged revealing that H_2_S carries out physiological functions by targeting proteins, enzymes, and transcription factors through a post-translational modification known as persulfidation. This review article provides a critical overview of the current state of the literature addressing the role of H_2_S in obesity-associated metabolic disturbances, with particular emphasis on its mechanisms of action in obesity, diabetes, non-alcoholic fatty liver disease (NAFLD), and cardiovascular diseases.

## 1. Introduction

Gasotransmitters are small molecules of endogenous gas that have the noticeable ability to diffuse into cells to interact with their targets, inducing an array of intracellular signaling and pathophysiological responses [1,2]. Hydrogen sulfide is the most recent addition to the gasotransmitters family, the first two being nitric oxide (NO) and carbon monoxide (CO) [3].

Instead of binding to plasma membrane receptors, the high solubility of gasotransmitters in lipids allows them to penetrate cell membranes without requiring a specific transporter or receptor. Gasotransmitters are generated endogenously by specific enzymes and can generate various functions at physiologically relevant concentrations by targeting specific cellular and molecular targets [4]. A gaseous substance is not readily stored in vesicular structures and so must be resynthesized as needed. This implies that the biosynthetic enzymes must be subject to tightly regulated mechanisms [5]. Abnormal generation and metabolism of these gasotransmitters have been extensively demonstrated to affect diverse biological processes, such as vascular biology, immune functions, cellular survival, metabolism, longevity, development, and stress resistance [4].

Perhaps the most remarkably unique feature of gasotransmitters relates to the molecular mechanisms whereby they signal to their targets. Gasotransmitters chemically modify intracellular proteins, thus affecting cellular metabolism in a more immediate fashion than other signal transduction mechanisms [5].

H_2_S has been traditionally considered only as a toxic agent for living organisms [6]. Nowadays, it is considered a gaseous mediator that plays important regulatory roles in innate immunity and inflammatory responses, impacting the development of cardiovascular and metabolic disorders [7,8,9,10,11]. Dysfunctional tissue H_2_S metabolism is increasingly implicated in different pathologies, from cardiovascular [12,13] and neurodegenerative diseases [14,15,16] to cancer [17,18]. There is emerging evidence supporting the importance of H_2_S in the pathophysiology of obesity, type 2 diabetes, NAFLD, and cardiovascular diseases. Understanding the precise mechanisms that control H_2_S homeostasis and their dysregulation is a major research focus. Despite decades of molecular and cellular studies on the enzymatic systems involved in H_2_S synthesis and breakdown, it appears at times that this field of biology is still in its infancy, with new targets of H_2_S and related species constantly being identified and new mechanistic details being revealed. In this review, we provide an overview of the current state of the literature about H_2_S in the context of obesity, diabetes, NAFLD, and cardiovascular diseases.

## 2. Biosynthesis of H_2_S

Hydrogen sulfide biosynthesis has been identified in a variety of mammalian tissues via enzymatic and non-enzymatic pathways [17]. In enzymatic biosynthesis, the endogenous generation of H_2_S from L-cysteine in the cytosol of cells is mainly mediated by two pyridoxal-5′-phosphate (PLP)-dependent enzymes known as cystathionine β-synthase (CBS) [19] and cystathionine γ-lyase (CTH or CSE) [20]. H_2_S is also produced by L-cysteine aminotransferase (CAT) and 3-mercapto-pyruvate sulfurtransferase (MPST) in the cytosol and mitochondria [21]. The expression of these enzymes is tissue-specific; in some tissues, CBS, CTH and MPST are all needed for the generation of H_2_S, whereas in others one enzyme serves this function. A small portion of endogenous H_2_S is derived via non-enzymatic reduction sulfur species, which are present in certain metabolites [17].

### 2.1. Enzymatic Synthesis of H_2_S

**Figure 1:**
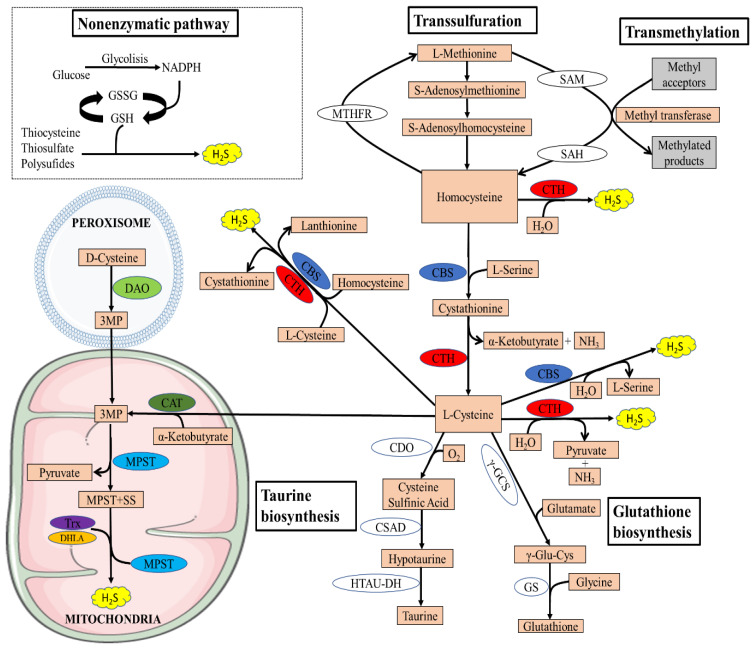
Overview of the transsulfuration pathway. There are four enzymatic pathways for the biosynthesis of H_2_S, including CBS, CSE, MPST coupled with CAT, and MPST coupled with DAO. Non-enzymatic H_2_S generation occurs in the presence of reducing equivalents such as NADPH and NADH, reactive sulfur species in persulfides, thiosulfate, and polysulfides that are reduced into H_2_S and other metabolites. The transsulfuration pathway intersects with the transmethylation pathway at homocysteine. Methionine is an indispensable amino acid and is transmethylated intracellularly via S-adenosylmethionine (SAM), an important methyl donor for most biological methylation reactions, producing S-adenosylhomocysteine (SAH) in this process, and is then hydrolyzed to homocysteine. Homocysteine can be remethylated back to methionine by N5,N10-methylenetetrahydrofolate reductase (MTHFR). The cysteine generated by the pathway can be conducted into GSH synthesis by the action of the enzymes γ-glutamyl cysteine synthetase (γ-GCS) and glutathione synthetase (GS) or converted to other sulfur-containing molecules such as taurine. Taurine is generated by the action of three enzymes, CDO, cysteine sulfinic acid decarboxylase (CSAD), and hypotaurine dehydrogenase (HTAU-DH).

The transsulfuration pathway plays a central role in sulfur metabolism and redox regulation in cells. The pathway leads to the generation of several sulfur metabolites, which include L-cysteine, glutathione (GSH), taurine, and the gaseous signaling molecule hydrogen sulfide [22]. In mammals, the pathway involves the transfer of sulfur from homocysteine to cysteine via cystathionine and is the only route for the biosynthesis of cysteine. Homocysteine, which is derived from dietary methionine, is converted to cystathionine by cystathionine β-synthase (CBS), which is acted on by cystathionine γ-lyase (CTH) to generate L-cysteine [22] (Figure 1).

In addition to its essential role in protein synthesis, cysteine is also a component of the major antioxidant GSH and is a potent antioxidant itself [23]. Disruption of cysteine and GSH metabolism has been frequently linked to aberrant redox homeostasis and neurodegeneration [23,24]. Both CTH and CBS play important roles in the regulation of redox balance. It has been reported that approximately 50% of the cysteine generated by the transsulfuration pathway is utilized for GSH biosynthesis in hepatic cells [25,26]. Cysteine is also the precursor of the gaseous signaling molecule hydrogen sulfide and other sulfur metabolites [27,28]. In addition to GSH and H_2_S, cysteine is converted to the sulfur-containing molecule taurine by the action of the enzyme cysteine dioxygenase (CDO) to form cysteinensulfinic acid, which can then be decarboxylated to hypotaurine by cysteine sulfinic acid decarboxylase, and the hypotaurine generated can be oxidized to taurine [29]. Since CDO acts directly on cysteine, it can modulate H_2_S production by influencing substrate availability. Mice lacking CDO show an elevated cysteine and H_2_S production capacity [30,31]. Taurine plays a role in osmoregulation, immunomodulation, neuromodulation, Ca^2+^ homeostasis, and ocular function and possesses antioxidant and anti-inflammatory effects [32]. The transsulfuration pathway is intimately linked to the transmethylation pathway via homocysteine, which can be remethylated to generate methionine or can be irreversibly converted to cysteine (Figure 1).

Recently, an alternative enzymatic pathway to the transsulfuration pathway has been identified for the enzymatic generation of H_2_S within mitochondria, known as the 3-mercaptopyruvate pathway. The pathway requires two enzymes, 3-mercaptopyruvate sulfurtransferase and the PLP-dependent enzyme cysteine aminotransferase (CAT). 3-mercaptopyruvate (3MP) is produced by CAT from L-cysteine and α-ketoglutarate [21]. Thereafter, MPST transfers a sulfur atom from 3MP onto itself, which leads to the formation of a persulfide, MPST-SS. H_2_S and MPST are then released from the persulfideof MPST-SS in the presence of reductants such as thioredoxin (TRX) and dihydrolipoic acid (DHLA) [33]. Recently, another source of 3MP was found in mammals by Shibuya et al.: D-cysteine [34]. Specifically, D-cysteine is transformed into 3MP by peroxisome-located d-amino acid oxidase (DAO). Metabolite exchanges between peroxisome and mitochondria can import 3MP into mitochondria, where it is further catalyzed into H_2_S by MPST. Because of the exclusive location of DAO in the brain and kidney, this H_2_S-generating pathway is currently believed to exist only in these two organs [17].

### 2.2. Non-Enzymatic Synthesis of H_2_S

Several cases of the non-enzymatic production of H_2_S have been reported, and these are suspected to represent a small proportion of all generated endogenous H_2_S. It is postulated that coordinated activities of PLP and iron (Fe^3+^) catalyze the generation of H_2_S using cysteine as a substrate, in a non-enzymatic manner in specific circumstances. Regulation of H_2_S production via this pathway may contribute to the pathophysiology of conditions with iron dysregulation such as hemolysis, iron overload, and hemorrhagic disorders [35].

H_2_S can also be generated from sulfane sulfur via non-enzymatical reduction in the presence of an endogenous reductant, such as nicotinamide adenine dinucleotide phosphate (NADPH) and nicotinamide adenine dinucleotide (NADH), which are supplied by oxidation of glucose via glycolysis or from phosphogluconate via NADPH oxidase [36]. In the presence of such reductants, reactive sulfur species in persulfides, thiosulfate, and polysulfides can be reduced into H_2_S and other metabolites [37]. Essentially, all the components of this non-enzymatic route are available in mammals, including reducible sulfur, suggesting the necessity of this pathway in mammalian systems. In accordance with this, hyperglycemia is demonstrated to promote H_2_S generation by enhancing this pathway [38].

## 3. The Relationship between H_2_S and Obesity

Obesity is characterized by the excessive accumulation and storage of fat in the body (regionally, globally, or both) that may be harmful to health and is defined by a body mass index (BMI) of 30 kg/m^2^ or greater, being considered morbidly obesity when BMI is over 35 kg/m^2^.

The epidemic of obesity presents a serious threat to human health around the world. The worldwide prevalence of obesity has increased dramatically over the past 30 years, fueled by economic growth, industrialization, mechanized transport, urbanization, an increasingly sedentary lifestyle, and a nutritional transition to processed foods and high-calorie diets [39]. According to the World Health Organization (WHO), 30% of Americans and 10%–20% of Europeans are obese, and on estimate more than 1.9 billion adults worldwide are overweight. High body mass carries with it an increased risk of the development of a number of serious cardiovascular and metabolic diseases, such as type 2 diabetes, hypertension, dyslipidemia, stroke, osteoarthritis, as well as several different forms of cancer [40]. There is emerging evidence supporting the importance of H_2_S in the pathophysiology of obesity.

### 3.1. The Importance of H_2_S in Obesity

Few studies have evaluated circulating sulfide in humans, with discrepant results. Whiteman et al. were the first ones to demonstrate the involvement of H_2_S in obesity [41]. These authors found that plasma H_2_S levels were significantly decreased in non-obese individuals with type-2 diabetes and in overweight participants with altered glucose [41]. However, the mechanisms that mediate the loss of H_2_S were not elucidated in that early study.

Additionally, other animal studies have demonstrated that exogenous H_2_S administration led to increased insulin sensitivity and improved glucose tolerance after a high-fat diet was fed to mice in parallel to weight gain [42,43]. Supplementation with H_2_S donors or increasing endogenous H_2_S biosynthesis was sufficient to stimulate fat mass accumulation in mice and fruit flies, whereas the depletion of endogenous H_2_S biosynthesis prevented high fat diet-induced fat mass (HFD-induced fat mass) [43,44]. Later, Alkhouri et al. reported that the H_2_S concentration in exhaled air was higher by 1/3 in obese children compared to lean children [45].

Recently, Comas and colleagues demonstrated that serum sulfide concentrations were increased in subjects with morbid obesity in proportion to fat mass [46]. Longitudinally, weight gain resulted in increased serum sulfide concentration, whereas weight loss had opposite effects, being the percent change in serum sulfide positively correlated with the percent change in BMI and waist circumference [46]. Ren and colleagues demonstrated that a milk protein concentrate diet prevents obesity induced by HFD in Sprague Dawley rats. This protection against obesity was associated with increased transsulfuration pathway and plasma H_2_S levels, which in turn maintained redox homeostasis and reduced lipid disorders induced by HFD [47].

Due to the lack of standardized methodology and variation in models and study cohorts, we see contradicting reports about H_2_S in obesity (Table 1). Depending on the technique used to measure sulfide, reported circulating levels of sulfide vary widely in obese and lean individuals. Furthermore, more human and animal studies are needed to fully comprehend the physiological and pathophysiological roles of H_2_S in obesity.

### 3.2. Persulfidation Might Prevent the Negative Effects of Obesity-Associated Oxidative Stress

Signaling by H_2_S is proposed to occur via persulfidation, a posttranslational modification of cysteine residues (RSH) to persulfides (RSSH) [48], thought to be one of its main beneficial mechanisms of action [28]. Like other posttranslational modifications, persulfidation potentially alters a protein’s structure, function, stability, and/or macromolecular interactions [48,49,50]. Nonetheless, due to their enhanced nucleophilicity, persulfides react readily with reactive oxygen species (ROS), whereas H_2_S itself is a poor ROS scavenger. When exposed to ROS, proteins undergo oxidation to form sulfenic acids (P-SOH), sulfinic acids (P-SO_2_H), and sulphonic acids (P-SO_3_H), which cause the irreversible inactivation of the protein [51].

The detrimental actions of ROS are well-known. ROS are physiologically important, acting as second messengers in cell signaling, and they also play a pivotal role in cellular homeostasis [52,53] and are associated with cellular damage by oxidizing cellular constituents such as proteins, lipids, and DNA [54]. These physiological effects are mainly mediated by changes in the redox state of crucial intracellular and/or surface thiols. In the last few years, several studies have shown a strong association between obesity and altered redox state, consistent with the idea that an increased caloric intake and/or obesity are associated with a pro-oxidant environment and increased oxidative damage [55,56,57,58,59]. A lot of evidence has shown that obesity is a state of chronic oxidative stress, although it is not completely understood if the alteration in redox balance is a trigger rather than a result of obesity [57,60,61,62,63].

**Figure 2:**
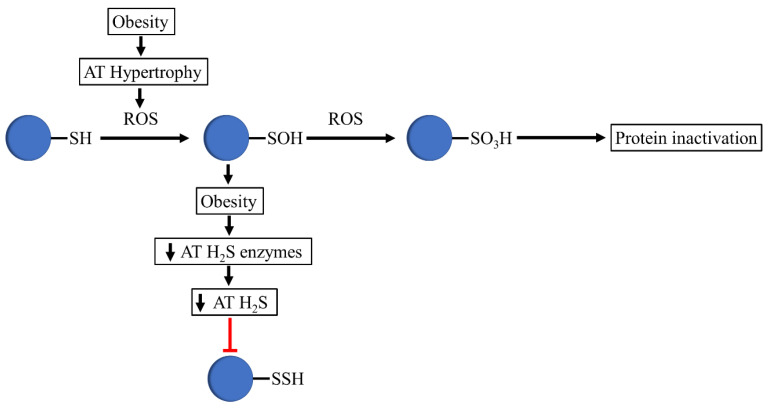
Proposed mechanism that occurs in thiols in obesogenic conditions. In obesity, adipose tissue hypertrophy is associated with increased ROS levels, which oxidizes protein thiols to sulfenic acids. The decreased expression of H_2_S-synthesizing enzymes and H_2_S production in adipose tissue avoids the formation of persulfides by sulfenic acid. The oxidizing environment of obesity mediates the conversion of sulfenic acids to sulfonate, altering the integrity and activity of relevant adipose-tissue-related proteins.

Given the fact that adipose tissue expansion during the progression of obesity can result in the excess production of toxic radical species that can cause the generation of oxidative stress through ROS, it is tempting to speculate that increased ROS in obesity leads to reduced persulfidation, resulting in cysteine thiol overoxidation, altering the integrity and activity of relevant adipose-tissue-related proteins (Figure 2).

Aside from decreased persulfidation in obesity due to ROS, alternative mechanisms have also been proposed. Dietary restriction (DR) regimens are known to reduce adiposity. Hine et al. reported that dietary restriction of sulfur amino acids (SAAs) methionine (Met) and cysteine (Cys) and decreased mTORC1 activation led to the increased expression of CTH, resulting in increased H_2_S production in the liver, and an increased lifespan [9]. Methionine and cysteine are considered to be the principal SAAs in the diet because they are incorporated into proteins. Elevation of plasma SAAs is generally associated with an unfavorable lipid profile, and plasma total cysteine is independently associated with body mass index [64]. Another study reported that higher plasma total cysteine was associated with an increased risk of obesity and IR in Hispanic children and adolescents [65]. These studies suggest that a pro-oxidant environment and increased oxidative damage, paired with increased SAAs in obesity, lead to decreased transsulfuration pathway (TSP) activity and decreased H_2_S, which could result in decreased persulfidation in obesity.

Aging is known to be associated with decreased persulfidated protective pools, associated with the loss of protein expression of the three H_2_S-producing enzymes [66]. Hine et al. showed that hepatic-produced H_2_S was elevated in long-lived hypopituitary mouse models, and that the thyroid hormone (TH) and growth hormone (GH) negatively regulate hepatic H_2_S via the repression of CTH [67]. In addition to the onset of age-related diseases in senior adults, there are some conserved aging phenotypes in human and animals—redox imbalance, mitochondrial dysfunction, increased apoptosis, cellular senescence, insufficient autophagy, and increased inflammation [68]. Similar to aging, obesity and excess calorie intake appear to perpetuate the onset of age-related diseases through similar mechanisms [69]. These findings indicate that obesity accelerates aging, allowing for cysteine overoxidation and reduced persulfidation.

Supporting the importance of H_2_S and persulfidation in the physiology of adipose tissue (Figure 2), Comas et al. recently demonstrated that proteins required for an appropriate adipogenesis and adipose tissue functionality present higher persulfidation levels in adipocytes in comparison to preadipocytes, sustaining the idea that persulfidation preserves the function of adipogenesis-related proteins [70].

Altogether, the evidence collectively suggests that obesity might be associated with reduced protein persulfidation, paving the way to cysteine overoxidation and impairing the activity and function of adipogenesis-related proteins.

## 4. H_2_S in Adipose Tissue

Adipose tissue is one of the most abundant organs in the human body, but for a long time it has been given little importance and it was considered only as a passive energy storage site [71]. The worldwide epidemic of obesity and type 2 diabetes has greatly increased interest in the biology and physiology of adipose tissues. It is now known that apart from the energy reservoir function, adipose tissue functions as a thermal insulator, mechanical shock absorber, and more importantly as an endocrine organ [72].

Feng et al. first identified the endogenous CTH and CBS gene expression pathways in adipose tissue, and suggested CTH as the primary pathway of H_2_S generation in adipose tissue [73]. The same year, Fang et al. also demonstrated that CTH protein expression and endogenous H_2_S production in rat perivascular adipose tissue were detectable and that the endogenous H_2_S generated was predominantly CTH-catalyzed [74]. Subsequent studies confirmed that CBS, CTH, and MPST genes were expressed in adipose tissue depots [75,76], and suggested that H_2_S affects diverse metabolisms that take place in the adipose tissue, such as lipid, glucose, and mitochondrial metabolism. H_2_S is also involved in the regulation of inflammatory and oxidative stress-associated responses in adipose tissue through adipokine and antioxidant control [77]. Studies of an in vitro 3T3-L1 mouse cell line model pointed to a possible role of H_2_S in adipocyte differentiation through the modulation of peroxisome proliferator-activated receptor gamma (PPARγ) activity [43,44,78]. The overexpression of the H_2_S generation enzyme CTH and the administration of the H_2_S donor sodium hydrosulfide (NaHS) to 3T3-L1 cells in an environment of high glucose restored adiponectin secretion and decreased the secretion of proinflammatory cytokines [79]. Recently, Comas et al. first reported the relevance of H_2_S and human adipose tissue physiology in the context of obesity [70]. Experiments in human adipose tissue explants and in isolated preadipocytes demonstrated that exogenous H_2_S or the activation of endogenous H_2_S biosynthesis resulted in increased adipogenesis, insulin action, sirtuin deacetylase, and PPARγ transcriptional activity, whereas inhibition through chemical compound or gene knockdown of one of the H_2_S-generating enzymes (CTH, CBS, MPST) led to altered adipocyte differentiation, cellular senescence, and increased inflammation. In morbidly obese subjects, reduced gene expression of H_2_S-synthesising enzymes in visceral and subcutaneous adipose tissue depots was reported, whereas weight loss interventions improved the expression of these enzymes. In human preadipocytes, the expression of CTH, CBS, and MPST genes and hydrogen sulfide production were dramatically increased during adipocyte differentiation [70]. In addition, a recent study demonstrated the relevance of selenium-binding-protein 1 (SELENBP1) in 3T3-L1 adipocyte differentiation through the modulation of cellular H_2_S levels and the expression of H_2_S-producing enzymes [80].

### 4.1. H_2_S and Lipid Metabolism

The effect of H_2_S on the regulation of lipid metabolism in adipose tissue is controversial. In primary adipocytes, inhibition of the CTH/H_2_S system was achieved with stimulated adipocyte lipolysis by means of increased activation of the PKA-perilipin1/hormone sensitive lipase (HSL) pathway, whereas GYY4137 or L-cysteine reversed this effect. In adipose tissue of normal chow and HFD mice, the CTH inhibitor DL-propargylglycine (PAG or PPG) increased lipolysis, evidenced by elevated serum glycerol, whereas H_2_S donors lowered lipolysis only in HFD mice [42]. Different studies have reported the use of slow hydrogen sulfide-releasing agents derived from garlic with effects on lipid metabolism. In isolated human adipocytes and Wistar rats, diallyl sulfide (DAS) downregulated the mRNA and protein expression of lipolytic genes like hormone-sensitive lipase (HSL) and adipose triglyceride lipase (ATGL), whereas it upregulated the expression of lipogenic gens like PPARγ [81]. Aged black garlic (AGB) extract suppressed lipogenesis by reducing the expression of PPARγ, whereas it enhanced lipolysis by upregulating HSL phosphorylation at Ser563 and downregulating perilipin in mature 3T3-L1 adipocytes [82].

On the other hand, infusing Na_2_S in adipose tissue of rats resulted in increased glycerol and cAMP release in control and obese rats, whereas PAG partially reduced glycerol levels in obese rats, suggesting an H_2_S-cAMP-PKA lipolysis mechanism [77].

A mechanistic way by which hydrogen sulfide could regulate lipid metabolism is persulfidation. Ding et al. showed that H_2_S post-translationally modified perilipin 1 (Plin-1) via cysteine persulfidation, resulting in increased Plin1 activity, which blocks HSL translocation to lipid droplets, resulting in decreased lipolysis and promoting adipocyte lipid accumulation [83].

### 4.2. H_2_S and Adipose Tissue Inflammation

Obesity predisposes patients to a pro-inflammatory state via increased inflammatory mediators interleukin-6 (IL-6) and tumor necrosis factor-α (TNF-α), as well as reduced levels of adiponectin, which has a totally anti-inflammatory function [84]. In recent years, substantial basic scientific research has led to a reasonably clear understanding of the role of H_2_S as an inflammatory mediator implicated in different inflammatory conditions, such as acute pancreatitis, sepsis, joint inflammation, and chronic obstructive pulmonary disease (COPD) [85]. Nevertheless, our understanding of the molecular mechanisms by which adipose tissue H_2_S contributes to inflammation in the obesity context is still scarce.

Adipose tissue macrophages (ATMs) are able to adopt either a proinflammatory (M1) or an anti-inflammatory (M2) phenotype. During obesity, the proinflammatory M1 phenotype is predominant. Store-operated calcium entry (SOCE), causing an influx of Ca^2+^, occurs in macrophage polarization to the M1 phenotype [86]. Increased amounts of CSE mRNA and protein and reduced H_2_S concentrations were found in ATMs isolated from obese mice and RAW264.7 macrophages under inflammatory conditions. Nonetheless, the H_2_S production capacity was found to be markedly increased in the previous conditions, leading to the increased consumption of H_2_S and reducing its bioavailability in proinflammatory conditions. This decrease in the concentration of H_2_S was associated with increased Ca^2+^ entry through the amplification of SOCE activity, facilitating the proinflammatory M1 phenotype in obese adipose tissue [87].

Recently, we examined the role of H_2_S in the regulation of inflammation during adipogenesis. Treatment with slow-releasing H_2_S donor GYY4137 attenuated the negative effect of inflammation on adipogenesis in 3T3-L1 during differentiation [88].

Active research on the role of H_2_S in inflammation will unravel the pathophysiology of its actions in adipose tissue inflammatory conditions and may help to develop novel therapeutic approaches.

## 5. The Possible Role of H_2_S on Glucose Metabolism

Most human cells utilize glucose as the primary substrate, with cellular uptake requiring insulin. Insulin signaling is therefore critical for these tissues. However, a decrease in insulin sensitivity due to the disruption of various molecular pathways causes insulin resistance (IR) [89]. Insulin resistance is one of the main characteristics of pathological manifestations associated with type 2 diabetes mellitus. In general, several intrinsic and extrinsic cellular mechanisms have been identified, which display a cause-effect relationship between weight gain and peripheral insulin resistance [90]. Intrinsic cell-signaling pathways include mitochondrial dysfunction, oxidative stress, and endoplasmic reticulum (ER) stress, whereas alterations in adipokines and fatty acids levels and the presence of inflammation in metabolic tissue are the dominant extrinsic mechanisms that modulate insulin’s peripheral actions [90].

**Figure 3:**
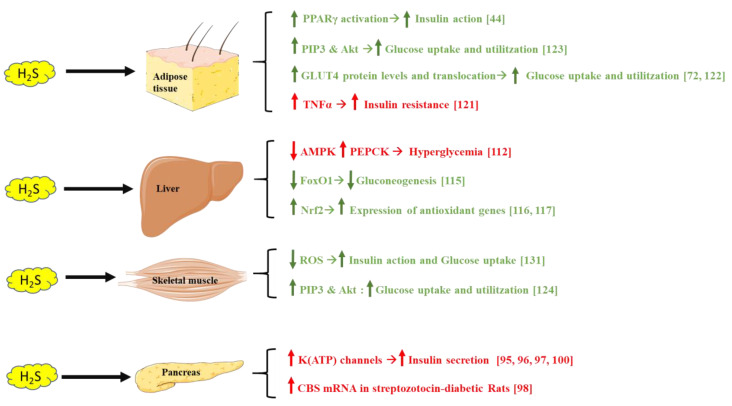
Different mechanisms showing how H_2_S affects adipose tissue, liver, skeletal muscle, and the pancreas, modulating insulin sensitivity and blood glucose levels. Green text indicates beneficial H_2_S effects on glucose metabolism, whereas red text indicates a negative impact on this process. Parts of the figure were drawn or modified by using pictures from Servier Medical Art (http://smart.servier.com/, accession: 13 April 2021), licensed under a Creative Commons Attribution 3.0 Unported License (https://creativecommons.org/licenses/by/3.0/, accession: 13 April 2021).

H_2_S has emerged as a regulator of glucose metabolism and energy homeostasis, through different mechanisms targeting the pancreas, liver, adipose tissue, and skeletal muscle (Figure 3).

### 5.1. H_2_S and the Pancreas

The pancreas is a complex gland, active in digestion and metabolism through the secretion of digestive enzymes, including the secretion of the blood sugar-lowering hormone insulin by β-cells and its opponent glucagon via the secretion of α-cells [91].

Insulin release from pancreatic β-cells is tightly regulated and allows the sensitive response of insulin levels to calorigenic nutrients in the body. Glucose, free fatty acids, and amino acids serve as fuel stimuli for insulin release, promoting insulin granule exocytosis. Additional hormonal factors influence the regulation pathway [92]. Insulin therapy is the most effective method of lowering blood glucose, thus pharmacological agents that augment insulin release are a key part of the treatment of diabetes.

All three enzymes, CBS, CTH, and MPST, are reported to be present at detectable mRNA and protein levels in rat pancreatic tissues or in cloned rat pancreatic β-cell lines (HIT-T15, INS-1E, and MIN6) [93,94,95].

At the start of the century, the pathophysiological implications of the CTH/H_2_S system in diabetes were reported. Yusuf et al. first demonstrated that CBS mRNA and H_2_S synthesis were increased in the pancreas from streptozotocin-induced diabetic rats [96]. Later, CSE and CBS gene expression were reported in mouse pancreatic acini [97]. In HIT-T15, a hamster insulin-secreting cell line, low levels of CBS were reported and no CTH mRNA transcripts were found [94]. In Zucker diabetic fatty (ZDF) rats, both CSE and CBS transcripts were detected in pancreatic islet tissues, with the expression level of CSE being significantly higher than that of CBS [98]. The expression of CBS protein in mouse pancreatic islets and a mouse β-cell line MIN6 was reported by Kaneko et al. [99,100]. INS-1E rat insulinoma cells expressed CTH, and DL-proparglycine mostly depleted H_2_S synthesis, indicating that CTH was the principal enzyme for H_2_S in INS-1E cells [93]. No MPST protein expression was detected by Western blot pancreases from C57BL/6J; however, later, strong levels of MPST protein were detected in the pancreas of C57/BL mice via immunohistochemistry using anti-rabbit IgG conjugated with HRP polymer and diaminobenzidine–hydrogen peroxide. Specifically, islets were strongly stained, whereas acinar wells were not stained [101]. Interestingly, Bronowicka-Adamska et al. reported for the first time the expression and activity of all enzymes involved in H_2_S production in the pancreas and suggested the most important role of MPST in Wistar Kyoto rats [102]. More human and animal studies are needed to elucidate the role of the interplay between H_2_S synthesizing enzymes, which seems to be a species-specific system.

### 5.2. H_2_S and the Liver

The human liver possesses the remarkable ability to produce glucose that is released to the systemic circulation and used by other tissues, particularly during periods of fasting. Hepatic glucose production derives from glycogen breakdown (glycogenolysis) and de novo synthesis of glucose (gluconeogenesis). In the fed state, plasma glucose is derived from the ingestion of nutrients, and the liver maintains normal plasma glucose levels by promoting glycogen synthesis and inhibiting gluconeogenesis [103]. All H_2_S-producing enzymes are expressed in the liver, which plays an important role in glucose and lipid homeostasis, xenobiotic metabolism, and antioxidant defense [104,105,106,107]. The published literature regarding the role of H_2_S in regulating glucose metabolism is controversial. Compared with non-diabetic rats, H_2_S production and CSE and CBS mRNA levels in the liver were increased in STZ diabetic rats, whereas insulin treatment reversed these effects [96]. Alternatively, H_2_S formation and CSE activity and protein expression in the liver were suppressed in STZ-induced type 1 diabetic rats [108]. Livers taken from insulin-sensitizer metformin-treated SJL mice (100 mg/kg b.w. per day) exhibited increased H_2_S concentrations [109].

In HepG2 hepatocytes, H_2_S has been shown to downregulate glucose uptake and glycogen storage, mediated through decreased AMP-activated protein kinase (AMPK) activation, resulting in increased activity of the gluconeogenic enzyme known as phosphoenolpyruvate carboxykinase (PEPCK), leading to hyperglycemia [110]. The same group also reported a stimulatory effect of H_2_S on liver glucose production under physiologic conditions [111]. On the other hand, Kundu et al. showed that H_2_S mitigates hyperglycemia in the liver by remodeling kinase B1-adenosine monophosphate-activated protein kinase signaling [112]. Another mechanism suggested by Guo et al. implies that CSE deficiency promoted liver gluconeogenesis in HepG2 via forkhead Box O1 (FoxO1) accumulation [113]. Other hepatoprotective effects of H_2_S donors were correlated with Nrf2 translocation and increased expression of antioxidant genes, which is regulated by kelch-like ECH-associated protein 1 (Keap1) persulfidation [114,115].

Further study will be needed to explore the regulatory mechanism of diabetes mellitus and its related conditions on the production of H_2_S in the liver.

### 5.3. H_2_S and Adipose Tissue

Adipose tissue plays a central role in regulating whole-body energy and glucose homeostasis through its subtle functions at both organ and systemic levels. On one hand, adipose tissue stores energy in the form of lipids and controls the lipid mobilization and distribution in the body. On the other hand, adipose tissue acts as an endocrine organ and produces numerous bioactive factors such as adipokines that communicate with other organs and modulate a range of metabolic pathways [116]. As explained, previous studies indicate that H_2_S plays an important role in adipose tissue, and all three enzymes are expressed in human adipose tissue [70].

Insulin exerts a critical control on anabolic responses in adipose tissue (AT) by stimulating glucose and free fatty acid uptake, inhibiting lipolysis and stimulating de novo fatty acid synthesis in adipocytes. In addition, insulin regulates AT growth and differentiation by enhancing the gene expression of various fat-specific transcription factors, including sterol regulatory element-binding protein 1 (SREBP-1c) and PPARγ [117]. Insulin increases glucose uptake in adipocytes by regulating the intracellular localization of glucose transporter 4 (GLUT4), the main glucose transporter involved in the insulin-regulated glucose transport from the cytosol compartment to the plasma membrane [118].

Various studies have shown that H_2_S regulates insulin sensitivity in adipocytes. In the adipose tissue of fructose induced-diabetic rats, the CTH/H_2_S system was upregulated and negatively associated with glucose uptake in AT, suggesting a pathological role of H_2_S in insulin resistance [73]. Huang et al. reported that H_2_S mediated TNF-α-stimulated insulin resistance, as the treatment of 3T3-L1 adipocytes with TNF-α lead to a deficiency in insulin-stimulated glucose consumption and uptake and an increase in endogenous H_2_S generation [119]. Chemical inhibition of CTH with PPG and β-cyano-L-alanine attenuated TNF-α-induced insulin resistance in 3T3-L1 adipocytes [119].

Other findings have suggested a beneficial role of H_2_S in glucose metabolism. Manna et al. showed a molecular mechanism in 3T3-L1 adipocytes by which H_2_S acts on 1,25-dihydroxyvitamin D3, upregulating GLUT4 protein levels and the translocation of GLUT4, essential for normal glucose metabolism [120]. Through persulfidation of PPARγ (Cys139) in 3T3-L1 cells and in the adipose tissue of HFD-mice, Cai et al. demonstrated that the CSE/H_2_S system attenuates insulin resistance and promotes the conversion of glucose into triglyceride storage through persulfidation [43]. Manna and Jain found that the administration of exogenous H_2_S using Na_2_S donors or stimulating the synthesis of H_2_S with L-cysteine increased phosphatidylinositol 3,4,5-trisphosphate (PIP3), which phosphorylated Akt and promoted glucose uptake and utilization in 3T3-L1 cells [121]. They also showed that exogenous H_2_S or L-cysteine supplementation increased insulin receptor substrate 1 (IRS1) phosphorylation and GLUT4 activation, resulting in upregulation of the metabolic actions of insulin and an improvement in glucose metabolism [121]. In another in vivo study, administration of exogenous H_2_S through NaHS or H_2_S gas solution promoted adipocytes to uptake glucose, thus reducing fasting blood glucose levels and increasing glucose tolerance [122]. H_2_S can protect adipocytes against high concentrations of glucose-induced adipocyte dysfunction, evidenced by the restored monocyte chemotactic protein (MCP)-1 and adiponectin secretion [79].

Recently Comas et al., showed that in ex vivo adipose tissue explants the stimulation of H_2_S synthesis via L-cysteine and pyridoxal 5′-phosphate (PLP) enhanced the expression of adipogenic genes in association with the insulin sensitivity, indicating a beneficial role of adipose tissue H_2_S in insulin pathway. In different human cohorts, insulin sensitivity was positively associated mainly with subcutaneous CTH and CBS, and weight loss resulted in increased CTH, CBS, and MPST mRNA, in parallel with improved insulin sensitivity [70]. In line with this, it is known that subcutaneous adipose tissue, VAT, is associated with insulin resistance, whereas SAT is associated with a decreased risk of insulin resistance [123]. In human adipocytes, exogenous administration of H_2_S using GYY4137 increased insulin action, whereas chemical inhibition with PPG attenuated insulin-induced Ser^473^Akt phosphorylation. Altogether, these findings suggest a mechanism by which H_2_S increases insulin action through the activation of PPARγ transcriptional activity in differentiated adipocytes [70]. Further study will be needed to explore the regulatory mechanism of adipose tissue H_2_S and glucose metabolism.

### 5.4. H_2_S and Skeletal Muscle

Skeletal muscle differentiation follows an organized sequence of events, including commitment, cell cycle withdrawal, and cell fusion to form multinucleated myotubes [124]. The skeletal muscle is the largest organ in the body by mass. It is also the regulator of glucose homeostasis, responsible for 80% of postprandial glucose uptake from the circulation. Skeletal muscle is essential for metabolism, both for its role in glucose uptake and its importance in exercise and metabolic disease [125]. Human skeletal muscles express significant amounts of CBS and CSE, whereas mouse skeletal muscles completely lack these enzymes [126]. A recent report suggested that all the three enzymes (CBS, CSE, MPST) were present in detectable levels in rat skeletal muscles [127]. Nonetheless, their expression is very low when compared to that of the liver and kidney. Only human skeletal muscles express CBS and CSE enzymes that are comparable to the expression levels in the liver in relative abundance [128].

Xue et al. provided evidence of the insulin-sensitizing effect of exogenous H_2_S administration (NaHS) in an in vitro model of myotubes (L6) and in vivo in Wistar Rats [122]. Using C2C12 mouse myotubes, exogenous H_2_S administration (NaHS) upregulated CSE expression and genes involved in GSH biosynthesis in parallel to increased H_2_S and glucose uptake and decreased ROS, whereas CSE knockdown had the opposite effects [129]. Chronic NaHS treatment (30 μmol·kg−1·day−1) in Goto-Kakizaki diabetic rats decreased fasting blood glucose, increased insulin sensitivity, and increased glucose tolerance with increased phosphorylation of PI3K and Akt in muscles [122]. Expression of CSE was declined at 20 weeks in skeletal muscle of db/db mice, compared to the control group, whereas administration of NaHS restores the expression of CSE at 20 weeks. In C2C12 myoblasts high glucose and palmitate and oleate reduced H_2_S and expression of CSE significantly [130]. It seems that H_2_S plays an important role in skeletal muscle, improving glucose homeostasis and increasing glucose uptake.

## 6. Implication of H_2_S in NAFLD

Non-alcoholic fatty liver disease (NAFLD) has become the principal cause of chronic liver disease worldwide, involving a spectrum of disturbances mainly characterized by fatty acid infiltration and fat deposition in the liver parenchyma, which can further progress to fibrosis, inflammation, and eventually develop into cirrhosis and hepatocellular carcinoma [131]. Identifying altered molecular pathways that trigger the onset and progression of the disease is a key point in ensuring early diagnoses and developing treatments.

In the liver, although the three H_2_S-generating enzymes are detectable, their roles in endogenous H_2_S generation are differently described [108,132,133]. It was found that CTH expression is about 60-fold greater than that of CBS in the murine liver [133]. Apart from endogenous hepatic synthesis, the liver is likely exposed to high levels of H_2_S exogenous sources as a consequence of its location, making the liver a key regulator of H_2_S levels by maintaining a high capacity for H_2_S clearance from the circulation [134].

**Figure 4:**
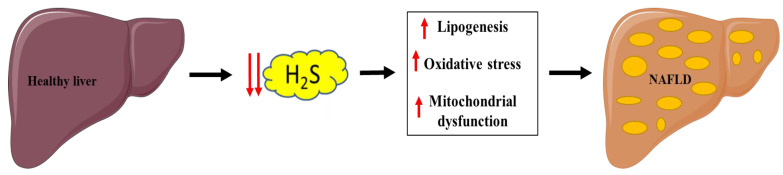
Link between decreased H_2_S production and NAFLD. Parts of the figure were drawn or modified by using pictures from Servier Medical Art (http://smart.servier.com/ accessed on 13 April 2021), licensed under a Creative Commons Attribution 3.0 Unported License (https://creativecommons.org/licenses/by/3.0/ accessed on 13 April 2021).

Pathological processes involved with NAFLD are linked with hepatic H_2_S pathways and include lipid metabolism dysfunction, oxidative stress, insulin resistance, inflammation, and mitochondrial dysfunction. Malfunction of hepatic H_2_S metabolism is involved in the pathogenesis of many liver diseases, such as hepatic fibrosis and cirrhosis [135] (Figure 4).

### 6.1. Mitochondrial and Lipid Metabolism Dysfunction

Since the first studies on NAFLD, many researchers have pointed out that it is primarily characterized by the presence of mitochondrial dysfunction [136]. Mitochondria play a pivotal role in hepatocyte metabolism, being the primary site for the oxidation of fatty acids and oxidative phosphorylation [137].

The CBS/CSE system, which may be regulated by several fatty acids, has been actively investigated in the pathogenesis of NAFLD and has been proposed as a potential therapeutic target for NAFLD [138].

Impaired endogenous H_2_S synthesis was reported to be associated with fatty liver induced by HFD feeding [106,139] or methionine and choline-deficient (MCD) diet feeding [104]. Treatment with H_2_S donor sodium hydrosulfide (NaHS), prevented nonalcoholic steatohepatitis (NASH) by reducing hepatic triglyceride and cholesterol levels in rodents fed with the MCD diet through increasing peroxisome proliferator-activated receptor alpha (PPARα) and reducing SREBP-1c gene expression in the liver, suggesting an antisteatogenic effect of H_2_S through the prevention of oxidative stress and inflammation [104]. In fatty livers of mice induced by HFD, administration of NaHS significantly reduced hypertriglyceridemia and improved NAFLD by activating liver autophagy flux and the AMPK-mTOR signaling pathway [139]. However, a recent study suggested that hepatic MPST promoted hepatic steatosis in HFD fed mice and the knockdown of MPST-stimulated H_2_S production, whereas overexpression of MPST markedly reduced the formation of H_2_S via inhibition of CSE/H_2_S and subsequent upregulation of SREBP-1c, c-Jun N-terminal kinase phosphorylation, and oxidative stress [138]. In addition, that study also demonstrated that the inhibition of MPST in L02 cells reduced free fatty acids (FFAs) and increased the expression of CTH and H_2_S [138].

Garlic oil derivates diallyl trisulfide/disulfide (DATS/DADS), used as H_2_S donors in mice, reduced fatty acid synthase (FAS) protein levels, which suppressed ethanol-induced hepatic mitochondrial dysfunction, suggesting the regulation of SREBP1, PPARα, and cytochrome p450 2E1 (CYP2E1) [140].

### 6.2. Oxidative Stress

Oxidative stress is an important pathophysiological mechanism in NAFLD pathogenesis. Growing evidence supports a key role for oxidative stress caused by the generation of ROS in the progression of NAFLD [141]. Disturbances in lipid metabolism lead to hepatic lipid accumulation, which affects various reactive oxygen species (ROS) generators, including mitochondria, the endoplasmic reticulum, and NADPH oxidase [142].

Oxidative stress involves molecular or cellular damage, resulting from deficiency of antioxidants and/or antioxidant enzyme systems, and disrupting the cellular reduction-oxidation balance [143]. The human body is equipped with a variety of antioxidants that serve to counterbalance the effect of oxidants, with H_2_S being one of the most important. In fact, H_2_S protects cells in various diseases by acting as an antioxidant that reduces excessive amounts of reactive oxygen species (ROS) and reactive nitrogen species (RNS) [144]. ROS are highly reactive molecules and can damage cell structures such as carbohydrates, nucleic acids, lipids, and proteins, and alter their functions [145].

Several studies have highlighted the role of H_2_S in cellular redox homeostasis, which can be summarized in two main mechanisms: i) modulating levels and activity of classic cellular antioxidants, such as glutathione (GSH) and thioredoxin (TRX) [146], and ii) increasing the activity or expression of the transcription nuclear factor (erythroid-derived 2)-like 2 (NRF2) and the histone deacetylase protein family of sirtuins (SIRTs), which in turn increase the expression of antioxidant enzymes (AOE) [114,115].

## 7. H_2_S on Cardiovascular Disease

The epidemiological tendency in the 20th century was accompanied by an increase in noncommunicable diseases, of which cardiovascular diseases (CVDs) are now the leading cause of mortality and morbidity worldwide [147]. CVDs are a cluster of diseases and injuries that affect the cardiovascular system, including the heart and blood vessels. Multiple factors are contributing to the epidemic of CVDs, including the rapid aging of the population, improved survival rate from other illnesses, progressive urbanization, increased calorie consumption, decreased physical activity, mental stress, and air pollution [148,149,150].

The main condition underlying CVD is atherosclerosis, a chronic inflammatory condition that involves different cell types, and several cytokines and adhesion molecules.

Atherosclerosis is a chronic and slowly progressive cardiovascular disease that affects arterial blood vessels by thickening and hardening as consequences of high plasma cholesterol concentrations, especially cholesterol in the form of low-density lipoprotein (LDL) [151]. In these processes, reactive oxygen species play a pivotal role, as they can cause the oxidation of lipids such as low-density lipoprotein (LDL) and polyunsaturated fatty acids that are deposited in the vascular wall, allowing plaque calcification, directly damaging cellular components, and further promoting inflammation by activating several pro-atherogenic transcriptional factors [152]. The accumulation of plaques consequentially narrows the arterial lumen and restricts the blood supply, causing atherosclerotic lesions, which through the action of several cytokines can rupture and lead to occlusion of the vascular lumen. Depending on the area of rupture, these can manifest as acute myocardial infractions or stroke or acute ischemia of any nearby organ [153].

Over the last few years, our understanding of atherosclerotic processes has vastly improved; however, there are still many mechanisms that have not been fully elucidated. One of the first reported physiological roles of H_2_S was its capacity to induce vasorelaxation, acting as a K(ATP) channel opener, and displaying important antihypertensive effects and cardioprotection properties [11,154,155,156]. In line with this, there is a growing body of evidence linking H_2_S with cardioprotection, including decreasing heart rate, exerting inotropic and proangiogenic effects, decreasing blood pressure, and causing vasodilation [157]. Reduced levels of H_2_S have been found in patients with acute or stable coronary artery disease [158], hypertension [159], and heart failure [160]. The impact of H_2_S on cardiovascular disease has been extensively updated and reviewed in recent studies [161,162,163,164,165].

### 7.1. Hypertension

In 2003, Zhang et al. demonstrated that H_2_S could exert beneficial effects on the pathogenesis of hypoxic pulmonary hypertension in rats [166]. Later, other groups using hypertensive experimental animal models showed that in rats which had spontaneous hypertension (SHR), the level of hydrogen sulfide and the gene expression and activity of CSE were reduced [167]. In SHR rats, reduced arterial pressure after systemic treatment with NaHS or after intracerebroventricular NaHS treatment were also reported [168].

CSE levels are reduced in the vessel wall of spontaneously hypertensive rats and both CSE and CBS are reduced in the resistance vessels of rats rendered hypertensive after dexamethasone treatment [167,169,170]. In addition, animals with salt-sensitive hypertension have lower levels of CBS [171]. A causal link between low CSE levels and high blood pressure was established following the observation that CSE KO mice exhibit hypertension [172].

Moreover, administration of sulfide salts or the slowly releasing H_2_S donor GYY4137 reduced blood pressure in a genetic model of hypertension, as well as in rats rendered hypertensive by angiotensin-II or L-NAME administration [173,174,175].

Other naturally occurring H_2_S donors such as garlic-derived compounds or isothiocyanates present in vegetables also demonstrated cardioprotective effects [176]. Allicin, a garlic-derived H_2_S donor, is known for lowering arterial blood pressure, among other cardiovascular protection systems [177]. Isothiocyanates, which are abundant in cruciferae like mustard and broccoli, are also considered important in the cardiovascular system. In the rat aortic ring, H_2_S derived from isothiocyanates was responsible for observed vasorelaxant effects [178]. Other synthetic H_2_S donors like thioamino acids, including thioglycine and thiovaline, were found to induce significant relaxation of the pre-contracted mouse aortic ring [179].

A great number of studies have been carried on the investigation of the modulation of blood pressure by means of exogenous and endogenous H_2_S. However, in humans, few studies have measured plasma H_2_S levels in association with hypertension. In a small cohort of patients with type 2 diabetes, lower plasma levels of H_2_S were associated with higher systolic blood pressure (SBP) and diastolic blood pressure (DBP) [41]. Reduced H_2_S plasma levels have been confirmed in a human cohort of hypertensive patients [180,181].

### 7.2. Atherosclerosis

There are strong indications that the loss of H_2_S contributes to the establishment and progression of the disease of atherosclerosis. Hydrogen sulfide shows an anti-atherosclerotic action by attenuating oxidative stress, reducing blood platelet activation, reducing the inflammation process, and preventing the proliferation of vascular smooth muscle cells [182,183,184].

Atherosclerosis is a lipoprotein-driven disease that leads to plaque formation at specific sites of the arterial tree through intimal inflammation, necrosis, fibrosis, and calcification [185]. H_2_S is known to reduce endothelial dysfunction by preventing plaque formation by counteracting the main aspects of atherosclerosis, such as oxidation, adhesion, proliferation, and calcification [165].

Increased oxidative stress can accelerate the development of atherosclerosis by means of endothelial cell dysfunction, adhesion of molecules, vascular smooth muscle proliferation and migration, platelet activation, lipid oxidation, matrix metalloproteinase activation, and alterations in vasomotor activity [186]. Several studies have highlighted the role of H_2_S in cellular redox homeostasis, which can be summarized in two main mechanisms: (i) modulating the levels and activity of classic cellular antioxidants, such as glutathione (GSH) and thioredoxin (TRX), and (ii) increasing the activity or expression of transcription nuclear factor (erythroid-derived 2)-like 2 (NRF2) and the histone deacetylase protein family of sirtuins (SIRTs), which in turn increase the expression of antioxidant enzymes.

H_2_S can ameliorate vascular calcification by decreasing the activation of alkaline phosphatase and reducing the gene expression of osteopontin [187].

Plasma H_2_S and aortic H_2_S levels were decreased in apolipoprotein E knockout (ApoE(−/−)) mice. CSE expression was reduced in oxidized LDL (Ox-LDL)-stimulated human aortic endothelial cells (HAEC) and in the aorta of high-fat-diet-induced ApoE(−/−) mice [188]. The expression of CSE is mainly upregulated in macrophages, foam cells, and myofibroblasts from the atherosclerotic lesions of human patients with carotid specimens. In mouse and human atherosclerosis, CSE expression is upregulated, but circulating and plasma levels of H_2_S are reduced, a phenomenon that can be attributed to the inhibition of CSE enzyme activity [189].

The main therapeutic strategy to lower or reverse atherosclerosis is statins, but the clinical benefits of statins are somewhat limited [190,191]. Surprisingly, recent reports have mentioned that treatment using statins can improve H_2_S production, but it is the lipophilic atorvastatin, rather than the hydrophilic pravastatin, that increases the net H_2_S production [192,193,194,195,196].

## 8. Conclusions

This review summarizes and discusses the literature concerning the roles and mechanisms of H_2_S on obesity-associated metabolic disturbances, including insulin resistance, NAFLD, and cardiovascular diseases. Impaired H_2_S metabolism is involved in obesity and adipose tissue disturbances; however, scientific understandings of the role of H_2_S and the mechanisms by which it is altered in obesity remain somewhat contradictory. Significant progress has been made in this field during recent years, but more research is warranted to address this discrepancy in the future. The majority of studies suggest that the deficiency of endogenous H_2_S synthesis is associated with NAFLD, due to altered pathways that include lipid metabolism dysfunction, oxidative stress, insulin resistance, inflammation, and mitochondrial dysfunction. However, the mechanisms involved are complex and are still incompletely understood. Based on the evidence, it is clear that H_2_S plays a protective role in cardiovascular diseases, including atherosclerosis. However, there are still many controversies surrounding the signaling pathways, beneficial roles, and harmful effects of H_2_S in cardiovascular diseases. H_2_S therapy has only entered a preliminary stage in terms of basic medical research or preclinical research. Nevertheless, based on the existing knowledge about the beneficial effects of H_2_S, the future of H_2_S as a potential therapy against obesity-associated metabolic disturbances is expected to be promising and exciting.

## Figures and Tables

**Table 1:** Findings present in the literature related to the reported circulating levels of sulfide in obesity.

Subjects	Sample	Methodology	Sulfide Levels (Mean)	Reference
Lean (*n* = 11) vs Overweight (*n* = 16)	Plasma	Zinc trap spectrophotometry	38.9 µM vs 22 µM	Whiteman et al. (2010)
Lean (*n* = 55) vs overweight/obese (*n* = 60) children	Exhaled breath	Selected-Ion Flow-Tube Mass Spectrometry	0.35 ppb vs 0.49 ppb	Alkhouri et al. (2014)
Non-obese (*n* = 54) vs Obese (*n* = 85)	Serum	Naphthalimide-based fluorescence probe	5.67 µM vs 10.08 µM	Comas et al. (2020)
Non-obese (*n* = 42) vs Obese (*n* = 40)	Serum	Naphthalimide-based fluorescence probe	7.35 µM vs 10.31 µM	Comas et al. (2020)
